# Preparation and Magnetic Properties of Cobalt-Doped FeMn_2_O_4_ Spinel Nanoparticles

**DOI:** 10.1186/s11671-021-03619-7

**Published:** 2021-11-04

**Authors:** Aleksandr A. Spivakov, Chun-Rong Lin, En-Szu Lin, Ying-Zhen Chen, Yaw-Teng Tseng

**Affiliations:** Department of Applied Physics, National Pingtung University, No. 1 Linsen Rd., Pingtung City, 900393 Taiwan

**Keywords:** FeMn_2_O_4_ nanoparticles, Spinel ferrite, Cobalt doping, Magnetic properties

## Abstract

Mixed-metal oxide nanoparticles have attracted great scientific interest since they find applications in many fields. However, the synthesis of size-controlled and composition-tuned mixed-metal oxide nanoparticles is a great challenge that complicates their study for practical application. In this study, Co-doped FeMn_2_O_4_ nanoparticles were synthesized by the solvothermal method in which the crystallization was carried out under autogenous pressure at temperatures of 190 °C for 24 h. The influence of Co doping on the evolution of the structural and magnetic properties was investigated by various methods. It was found from XRD data that crystallite size decreases from 9.1 to 4.4 nm with the increase in Co content, which is in good agreement with the results of TEM. Based on the results of magnetic measurements, it was found that the saturation magnetization first increases with an increase in the cobalt content and reaches its maximum value at *x* = 0.4, and a further increase in *x* leads to a decrease in the saturation magnetization. The influence of cation redistribution on the observed changes has been discussed.

## Introduction

Due to the unique magnetic, electrical, and other properties, spinel oxides have attracted great scientific interest and find practical applications in various fields, such as spintronic devices, data storage, supercapacitors, biomedicine, light absorption, environmental remediation, and so on [1–7]. One of the reasons for the wide variety of physicochemical properties of spinel oxides is their structure with the general chemical formula AB_2_O_4_ (where *A* and *B* are metal ions). Depending on the distribution of ions between the tetrahedral A and octahedral B sites, spinels are divided into three types: normal, inverse, and mixed spinels [8, 9], and the structural formula for a binary spinel may be written in the more accurate format: $\left( {A_{1 - i}^{p + } B_{i}^{q + } } \right)\left[ {A_{i}^{p + } B_{2 - i}^{q + } } \right]O_{4}^{2 - }$, where the tetrahedral and octahedral sublattices are denoted as () and [], respectively; *p* and *q*—valencies; ‘*i*’—the inversion parameter, which is 0 for normal, 1 for inverse, and 0 < *i* < 1 for mixed spinels. In addition, the substitution of cations in spinel oxides also significantly affects their physical properties and increases opportunities for their practical application [10–13].

The Mn_*x*_Fe_3−*x*_O_4_ system has attracted the attention of researchers for a long time [14–16] due to its physical properties depend on the composition, which increases the possible applications of this system [17–22]. At the manganese content *x* < 1.9, it crystallizes in a cubic structure, while at *x* > 1.9 it crystallizes in a tetragonal structure (for bulk and single crystals samples) [23], which originates from the orientation of the tetragonally distorted Mn^3+^O_6_ octahedra due to the Jahn–Teller effect [23–25]. Despite the wide variety of compositions of the Mn_*x*_Fe_3−*x*_O_4_ system, most studies have focused on the iron-rich region (with *x* ≤ 1), while the number of reports on the manganese-rich region is limited [26–28]. It has been shown that in the Mn-rich region the system forms in an inverse or a mixed spinel structure [29] and cation distribution can be expressed by two formulae: $\left( {{\text{Mn}}^{2 + } } \right)\left[ {{\text{Fe}}_{3 - x}^{3 + } {\text{Mn}}_{x - 1}^{3 + } } \right]{\text{O}}_{4}^{2 - }$ or $\left( {{\text{Mn}}_{1 - y}^{2 + } {\text{Fe}}_{y}^{3 + } } \right)\left[ {{\text{Fe}}_{z}^{3 + } {\text{Mn}}_{2 - x}^{3 + } {\text{Mn}}_{y}^{2 + } } \right]{\text{O}}_{4}^{2 - }$ (where *x* = *y* + *z*). In the present work, we report, for the first time, as far as we know, about the study of FeMn_2_O_4_ nanoparticles doped with cobalt, which were synthesized by the solvothermal method. The influence of the Co content on the structural and magnetic properties of the nanoparticles was investigated by various methods.

## Methods

### Synthesis of Co-Doped FeMn_2_O_4_ Nanoparticles

**Scheme 1:**
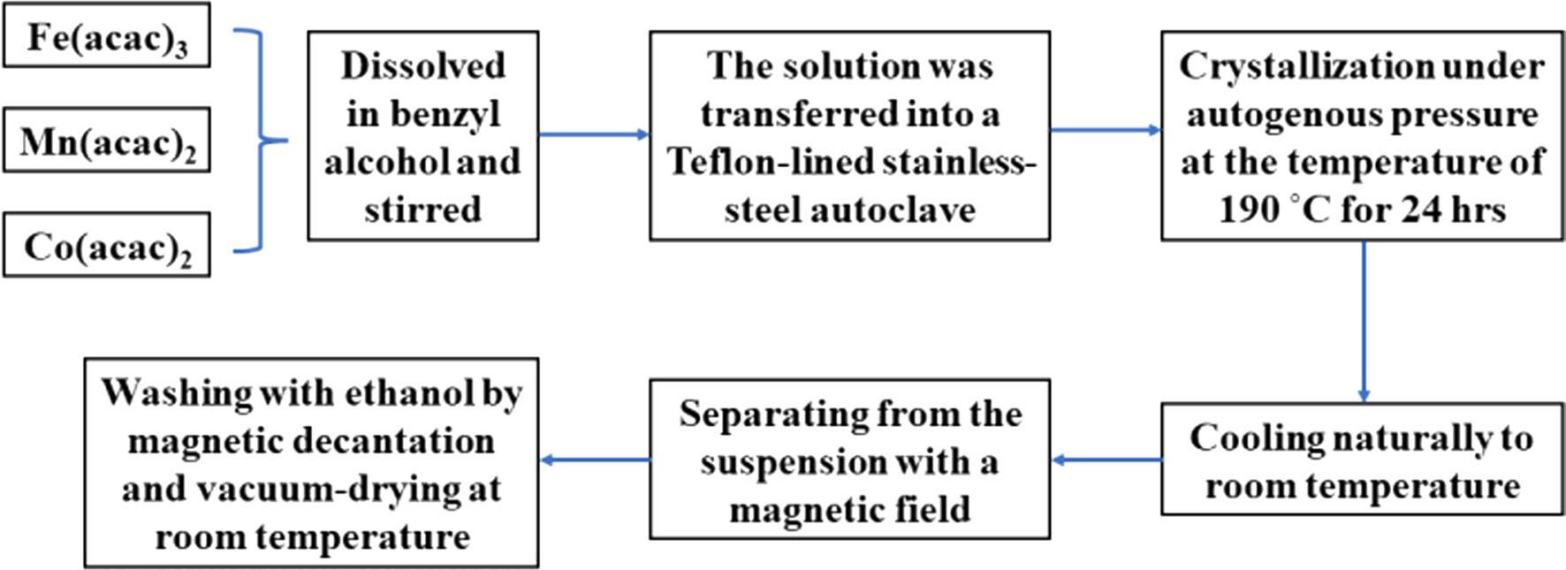
Flowchart for the synthesis of Co-doped FeMn_2_O_4_ nanoparticles

Samples of Fe(Mn_1−*x*_Co_*x*_)_2_O_4_ spinel nanoparticles were synthesized by the solvothermal method (Scheme 1). All the reagents were of analytical grade and were used without any further purification. The required quantities of Fe(acac)_3_, Mn(acac)_2_ and Co(acac)_2_ (see Table 1) were dissolved in benzyl alcohol. The resulting solutions were stirred thoroughly and then transferred into a 50 mL Teflon-lined stainless-steel autoclave to a filling capacity of 50%. The crystallization was carried out under autogenous pressure at the temperature of 190 °C for 24 h. Then, the autoclave was cooled naturally to room temperature, and the obtained nanoparticles can be separated from the suspension with a magnetic field. To remove the excess organic solvent and by-products completely, the products were washed several times with ethanol by magnetic decantation and vacuum-dried at room temperature.

**Table 1:** Composition, abbreviations of the sample names, and quantities of reagents required for the synthesis of the samples

Samples and their abbreviations	Fe(acac)_3_ (g)	Mn(acac)_2_ (g)	Co(acac)_2_ (g)	benzyl alcohol (mL)
FeMn_2_O_4_ (S1)	0.6102	0.8664	0	20
Fe(Mn_0.8_Co_0.2_)_2_O_4_ (S2)	0.6102	0.6932	0.176	20
Fe(Mn_0.6_Co_0.4_)_2_O_4_ (S3)	0.6102	0.52	0.3522	20
Fe(Mn_0.4_Co_0.6_)_2_O_4_ (S4)	0.6102	0.3466	0.5282	20
Fe(Mn_0.2_Co_0.8_)_2_O_4_ (S5)	0.6102	0.1732	0.7044	20
FeCo_2_O_4_ (S6)	0.6102	0	0.8804	20

### Characterization

The crystal structure and morphology of the nanoparticles were characterized by X-ray diffraction measurements using a Bruker D8 Advance diffractometer (Cu Kα radiation, 40 kV, 25 mA, *λ* = 1.5418 Å) and transmission electron microscopy (JEOL JEM-1230 microscope operated at an accelerating voltage of 80 kV). The ICP-MS analysis was carried out using high-resolution ICP-MS system Thermo Scientific ELEMENT XR. The Raman spectra were obtained using a Shamrock 750 spectrograph equipped with a CCD detector. The 533-nm line from the CW He–Ne randomly polarized laser was used for excitation. Magnetic properties were measured by a vibrating sample magnetometer (Lakeshore 7400 series VSM) in the applied field of *H* =  ± 17 kOe.

## Results and Discussions

**Fig. 1:**
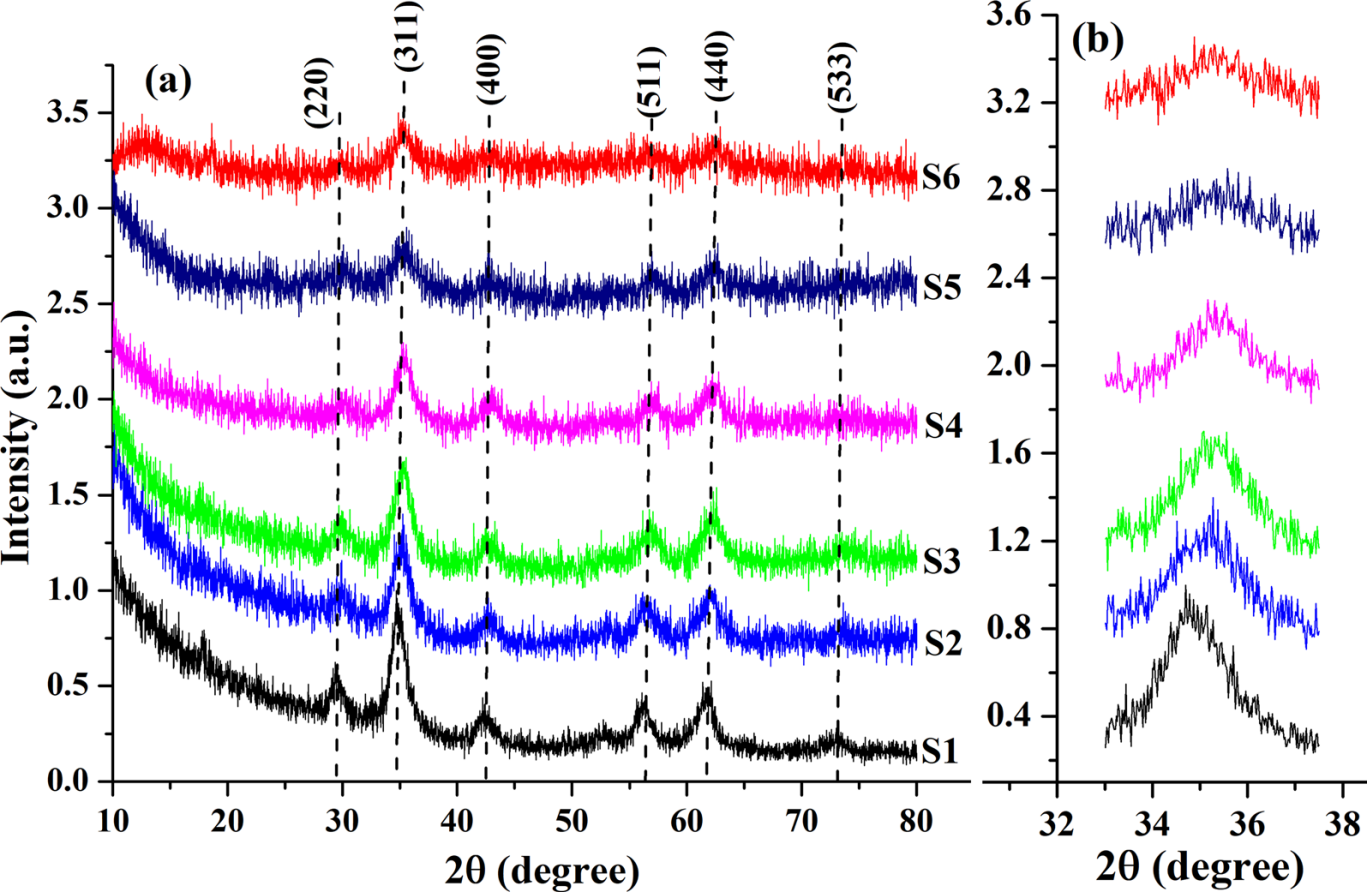
X-ray diffraction patterns of the Fe(Mn_1−*x*_Co_*x*_)_2_O_4_ nanoparticles (**a**) and shifting of (311) peak (**b**)

The XRD patterns of the samples with various concentrations of cobalt are shown in Fig. 1a. It can be seen that as the Mn content increased, the peaks in XRD spectra become narrower and sharper, which indicates an increase in the crystallite size of nanoparticles and their better crystallinity. The diffraction peaks at 29.4°, 34.9°, 42.4°, 56.4°, 61.7, and 73.1° correspond to the planes indexed (220), (311), (400), (511), (440), (533), respectively, and they are consistent with standard JCPDS Card No. 10–0319 of jacobsite ferrite with a face-centered cubic structure (space group $Fd\overline{3}m$). Although bulk samples crystallize in a tetragonal structure, a similar XRD patterns indicating the formation of a cubic structure was observed for FeMn_2_O_4_ nanoparticles [17, 18], which may be associated with the existence of a size-dependent phase transition in FeMn_2_O_4_ nanoparticles [30].

The average crystallite size (from the broadening of the most intensive peak (311)) and the lattice parameter of the synthesized samples were calculated in accordance with the relations (1) and (2), and the results are given in Table 1. The calculated values confirmed that crystallite size decreases with the increase in Co content from 9.1 nm (for the sample S1) to 4.4 nm (for the sample S6).

$$d_{XRD} = \frac{0.89\lambda }{{\beta \cos \theta }} \left( 1 \right);\;\;a = d_{hkl} \sqrt {h^{2} + k^{2} + l^{2} } \left( 2 \right)$$

where *λ*—the radiation wavelength (0.15418 nm for Cu Kα); *β*—the line broadening of a diffraction peak at angle *θ*; *d*_*hkl*_—inter planar distance; (*hkl*) are the Miller indices.

The results obtained revealed that the lattice parameter (‘a’) decreases from 8.52 to 8.37 as Co concentration increases. Besides, the data (Fig. 1b) show that with increasing Co content the position of (311) peak slightly shifts toward higher values of 2*θ*. This shift as well as the decrease in ‘a’ are related [31, 32] to the substitution of larger Mn ions (*r*_Mn_ = 0.645 Å) for Co ions (*r*_Co_ = 0.545 Å) on the octahedral sites.

ICP-MS analysis was performed to determine the actual composition of the synthesized samples. The results of the analysis showed that in the range 0 ≤ *x* ≤ 0.4 the actual compositions are in good agreement with expected ones, while in the range 0.4 < *x* ≤ 1 the actual compositions are slightly shifted toward lower values of *x* (see Table 2), indicating a slight loss of Co during synthesis of these samples.

**Table 2:** The actual compositions obtained from the results of ICP-MS, the average crystallite sizes calculated by the Scherrer equation (*d*_*XRD*_), and lattice constants (*a*) of the Co-doped FeMn_2_O_4_ nanoparticles

Expected composition	Actual composition	*d*_*XRD*_, nm	*a*, Å
FeMn_2_O_4_	FeMn_1.9_O_4_	9.1	8.52
Fe(Mn_0.8_Co_0.2_)_2_O_4_	Fe(Mn_0.8_Co_0.15_)_2_O_4_	8.5	8.47
Fe(Mn_0.6_Co_0.4_)_2_O_4_	Fe(Mn_0.6_Co_0.35_)_2_O_4_	7.6	8.45
Fe(Mn_0.4_Co_0.6_)_2_O_4_	Fe(Mn_0.35_Co_0.5_)_2_O_4_	6.5	8.41
Fe(Mn_0.2_Co_0.8_)_2_O_4_	Fe(Mn_0.2_Co_0.7_)_2_O_4_	5.3	8.4
FeCo_2_O_4_	FeCo_1.8_O_4_	4.4	8.37

**Fig. 2:**
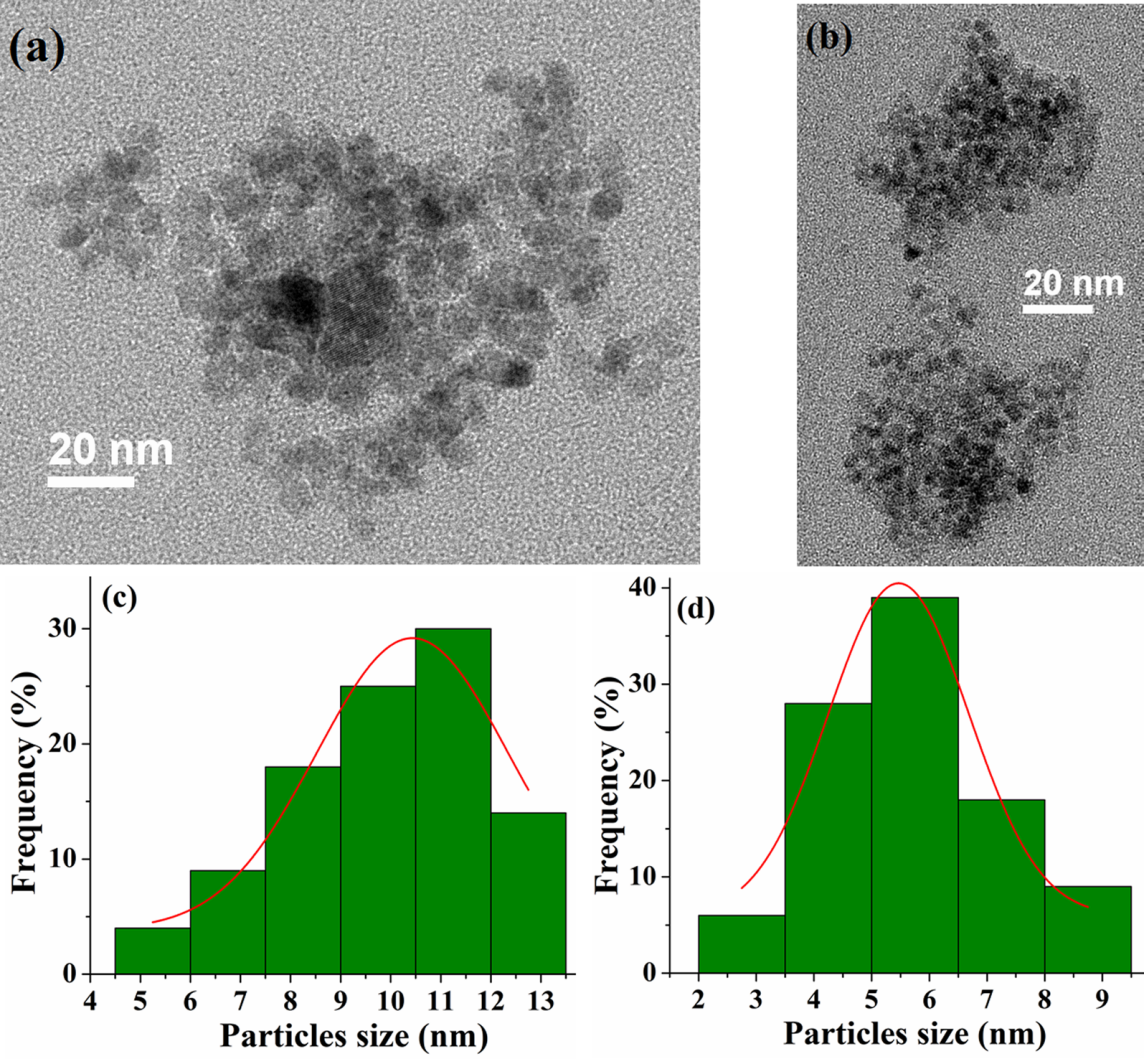
TEM micrographs of the samples and the histograms of the particle size distribution: (**a**), (**c**) for FeMn_2_O_4_ nanoparticles; (**b**), (**d**) for FeCo_1.8_O_4_ nanoparticles

TEM images for FeMn_2_O_4_ and FeCo_1.8_O_4_ samples are shown in Fig. 2 and demonstrate that particles uniform in size and have a spherical or quasi-spherical shape with a tendency to agglomerate. The agglomeration of the nanoparticles may be related to the influence of Van der Waals forces that dominates all other forces when the particle size is less than a few micrometers [33]. Figure 2c and d demonstrates the particle size distribution for the samples S1 and S6 with Gaussian fitting of the distribution. The average particle sizes are 10.5 ± 2 nm (*x* = 0) and 5.3 ± 1.5 (*x* = 0.9) nm, and these values are in good agreement with the results obtained by XRD.

**Fig. 3:**
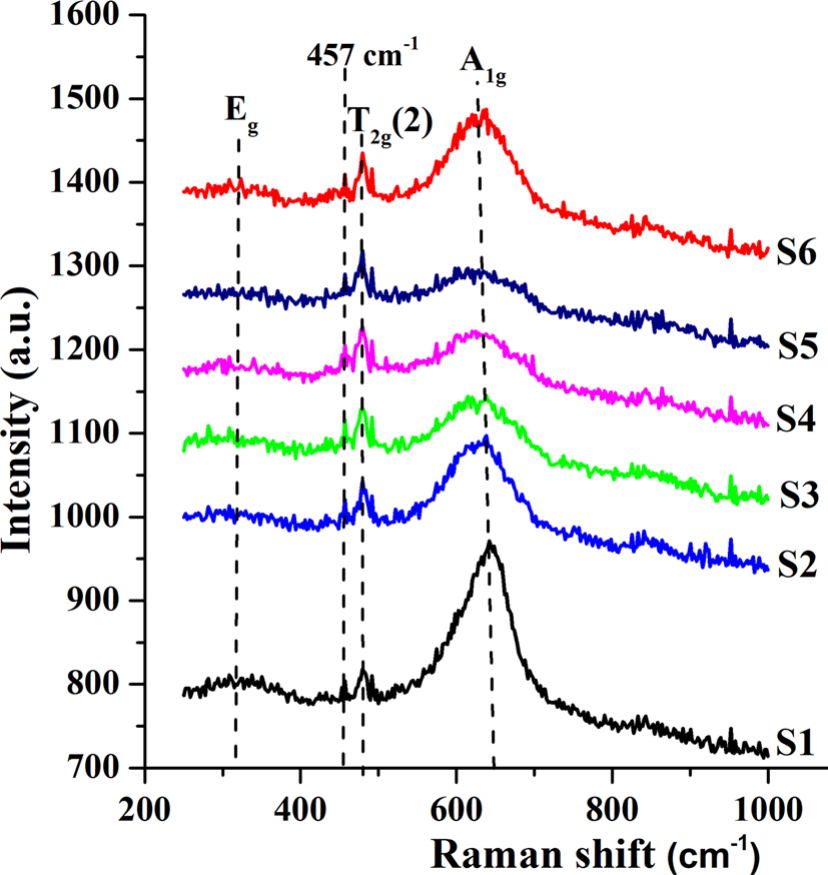
Room-temperature Raman spectra of the Fe(Mn_1−*x*_Co_*x*_)_2_O_4_ nanoparticles

The Raman spectra of Co-doped FeMn_2_O_4_ nanoparticles in the range of 250–1000 cm^−1^ are presented in Fig. 3. The XRD analysis revealed that the synthesized samples crystallized in a cubic structure and group theoretical analysis for space group $Fd\overline{3}m$ predict [34] five Raman active modes: *A*_1g_, *E*_g_, and three *T*_2g_. In our samples, only three major peaks were detected in Raman spectra: two intense at ~ 634 cm^−1^ and 479 cm^−1^ one weak at ~ 321 cm^−1^. Based on the previous studies of Raman spectra of spinel oxides [34, 35], it can be concluded that the Raman peaks correspond to the following modes: peak at ~ 634 cm^−1^ is due to *A*_1g_ mode involving symmetric stretching of oxygen atoms concerning the metal ions in tetrahedral AO_4_ groups. It can also be seen that the peak is broadened for the samples 0 ≤ *x* ≤ 0.9, which is related to the replacement of Mn^2+^ to Co^2+^ ions in tetrahedral sites leading to a redistribution of Mn/Co–O bonds and, as a consequence, broadening of *A*_1g_ peak. Two low-frequency modes at ~ 321 and ~ 479 cm^−1^ correspond to *E*_g_ and *T*_2g_(2) modes, respectively, and are related to metal ions involved in octahedral BO_6_ sites. The peak at ~ 457 cm^−1^ can be assigned phenyl ring deformation out-of-plane of benzyl alcohol [36], which was used in the synthesis process. Thus, the results of Raman spectroscopy confirmed the cubic structure of the synthesized nanoparticles.

**Fig. 4:**
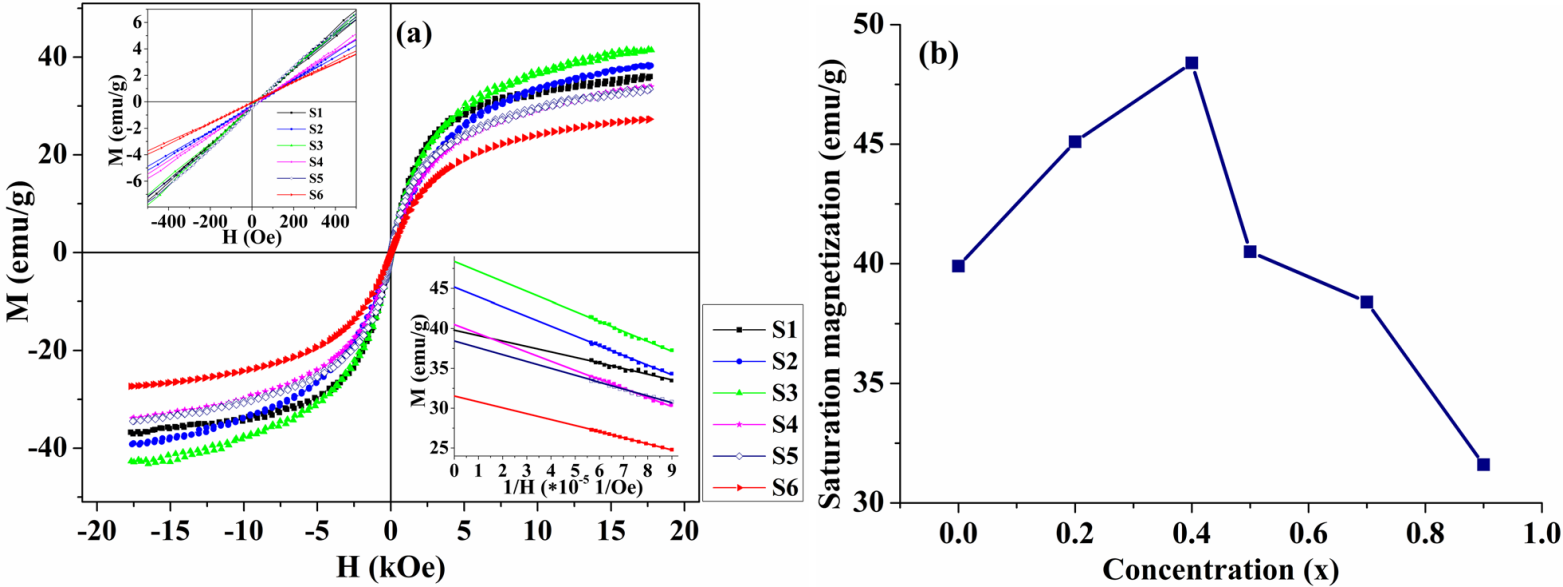
Magnetic hysteresis loops of the samples with 0 ≤ *x* ≤ 0.9 a) and concentration dependence of the saturation magnetization (b). The upper inset shows the hysteresis loops on an enlarged scale; The lower inset shows *M* versus 1/*H* curves in high magnetic fields

The magnetic hysteresis loops of the Fe(Mn_1-x_Co_x_)_2_O_4_ nanoparticles measured at room temperature are shown in Fig. 4a and b that presents a dependence of the saturation magnetization on cobalt concentration.

As can be seen from Fig. 4a, the magnetic hysteresis loops of the samples are S-like curves with zero remanent magnetization and coercivity, which indicates that all synthesized samples are superparamagnetic at room temperature. The values of the saturation magnetization obtained from the analysis of *M* versus 1/*H* curves are presented in Fig. 4b. It should be noted that the value of saturation magnetization for sample S6 is slightly lower than that reported in the literature (*M*_S_ = 40.5 emu/g) [37] for larger nanoparticles (*d*_*XRD*_ = 21.6 nm) which can be explained by the influence of the size effect on the magnetic properties. At the same time, the obtained value is higher than for coated FeCo_2_O_4_ nanoparticles (*M*_S_ = 22 emu/g; *d* ~ 40 nm) [17]. Thus, we can conclude that although the Raman measurements revealed a trace of benzyl alcohol, its presence on the surface of the synthesized nanoparticles is rather small and does not affect their magnetic properties.

The obtained results demonstrate that the saturation magnetization first increases with a corresponding increase in the Co content from 39.9 (*x* = 0) to 48.4 emu/g (*x* = 0.4) and with a further increase in x, the saturation magnetization decreases to 31.6 emu/g (*x* = 0.9). Since an atomic magnetic moment of Co^2+^ (3 µB) is less than magnetic moments of Mn^2+^ and Fe^3+^ (5 µB for both) [38, 39], it is expected the decrease in magnetization with the increase in Co content, which is in agreement with experimental results in the range of 0.4 < *x* ≤ 0.9. However, for the concentration range 0 ≤ *x* ≤ 0.4, an increase in the saturation magnetization is observed with increasing x, which can be explained by the redistribution of cations between tetrahedral and octahedral sites. In accordance with Néel’s two-sublattice theory, inter-sublattice interaction (*A*—*B*) is much stronger than the intra-sublattice interactions (*A*—*A* and *B*—*B*) and the net magnetization is proportional to the difference between the magnetic moment of tetrahedral (*M*_*A*_) and octahedral (*M*_*B*_) sites and is given by ${M}_{S}={M}_{B}-{M}_{A}$ [40]. It is assumed that at low concentration Co^2+^ ions push Fe^3+^ ions from tetrahedral to octahedral B sites, which leads to an increase in the octahedral magnetic moment due to an increase in Fe^3+^ ions and, as a result, an increase in the net magnetization.

## Conclusions

The effect of Co doping on the structural and magnetic properties of Fe(Mn_1-x_Co_x_)_2_O_4_ nanoparticles prepared by the solvothermal method was studied. The results of the structural analysis showed that particles are uniform in size and have spherical or quasi-spherical shapes, herewith with the increase in cobalt content, the average particle size decreases from 10.5 ± 2 nm (*x* = 0) to 5.3 ± 1.5 (*x* = 0.9) nm. Although bulk and single crystal samples of FeMn_2_O_4_ crystallize in a tetragonal structure, the results of XRD and Raman showed that the synthesized nanoparticles crystallized in a cubic structure, which may indicate the existence of a size-dependent phase transition in FeMn_2_O_4_. Magnetic measurements revealed the superparamagnetic nature of all samples at room temperature. It has been found that in the range of 0.4 < *x* ≤ 0.9 the saturation magnetization decreases, as expected. However, for the range of 0 ≤ *x* ≤ 0.4, an increase in the saturation magnetization is observed. Such behavior can be associated with the redistribution of Fe^3+^ ions between tetrahedral and octahedral sites.

## Data Availability

The raw and processed data required to reproduce these findings cannot be shared at this time as the data also forms part of an ongoing study. However, some data required to reproduce these results can be provided upon request by email: aleksandr.a.spivakov@gmail.com.
